# Child Consonant Harmony Revisited: The Role of Lexical Memory Constraints and Segment Repetition

**DOI:** 10.1177/00238309241297703

**Published:** 2024-12-03

**Authors:** Mitsuhiko Ota

**Affiliations:** School of Philosophy, Psychology and Language Sciences, University of Edinburgh, UK

**Keywords:** Children, consonant harmony, assimilation, word production, memory

## Abstract

Young children often produce non-target-like word forms in which non-adjacent consonants share a major place of articulation (e.g., [gɔgi] “doggy”). Termed child consonant harmony (CCH), this phenomenon has garnered considerable attention in the literature, primarily due to the apparent absence of analogous patterns in mature phonological systems. This study takes a close look at a potential account of CCH that is compatible with findings from adult word learning, serial recall, and phonological typology. According to this account, CCH is a response to memory pressure involved in remembering and retrieving multiple consonantal contrasts within a word. If this is the main motivation behind CCH, we would expect the resulting child forms to be biased toward full assimilation (i.e., consonant repetition) as it allows maximal reduction of phonolexical memory load. To test this prediction, children’s productions of target words containing consonants that differ in both major place and manner were analyzed using two data sources: a single session sample from 40 children aged 1–2 years learning English, French, Finnish, Japanese, or Mandarin; and longitudinal samples from seven English-learning children between 1 and 3 years of age. Prevalence of consonant repetitions was robustly evidenced in early child forms, especially in those produced for target words with the structure CVCV(C). The results suggest that early word production is shaped by constraints on phonolexical memory.

## 1 Introduction

### 1.1 Background

Few phenomena in early word production have attracted as much attention in the literature as child consonant harmony (CCH): children’s production of non-target-like word forms with apparent non-local assimilation of major place between two consonants (e.g., [gɔgi] *doggy*, [gʌk] *duck*) (Berg & Schade, 2000; Cruttenden, 1978; Fikkert & Levelt, 2008; Goad, 1997; Levelt, 1994; Menn, 1978; Pater & Werle, 2003; Y. Rose, 2001; Smith, 1973; Stoel-Gammon & Stemberger, 1994; Vihman, 1978; see a comprehensive review by Levelt, 2011). Researchers maintain interest in CCH primarily because it has no apparent counterpart in adult phonology. While many languages exhibit some form of non-local agreement between consonants, none mirror the major place of articulation changes characteristic of CCH (S. Rose, 2011; S. Rose & Walker, 2011). Furthermore, CCH contrasts sharply with the observed tendency in languages to avoid similar major places of articulation in CVC configurations, such as /. . .mip. . ./ and /. . .kug. . ./, where consonants share labial and dorsal places, respectively. Although not strictly prohibited, lexical roots containing such configurations are underrepresented across typologically diverse languages, suggesting a universal dispreference against transvocalic place-sharing consonants (Pozdniakov & Segerer, 2007). Much of the discussion surrounding CCH has revolved around the question of what drives CCH and why the effects of its underlying mechanisms are not visible in—and are even seemingly contradicted by—adult phonology.

In this study, I revisit the theoretical underpinnings and empirical evidence supporting an explanation for CCH that has garnered less attention. Initially proposed by Vihman in 1978, this account views CCH as a response to the memory pressures associated with the recall of multiple consonantal contrasts within a word. In the remaining sections of the introduction, I will review salient features of CCH, compare previous accounts, and demonstrate that viewing CCH as a product of phonolexical encoding and retrieval constraints aligns best with what is known about word learning, serial recall, and the typological patterns observed in adult phonology. A corollary of this account is that the assimilation of consonants in early word production should be biased toward maximal redundancy, manifesting as full assimilation or the repetition of identical consonants. The empirical contribution of this study is to examine the extent to which this prediction is supported by evidence based on transcribed spontaneous speech-production data.

### 1.2 Characteristics of CCH and previous accounts

There are some well-known characteristics of CCH, at least in relation to English (Levelt, 2011). The phenomenon typically “targets” a coronal (i.e., the consonant that undergoes change is more likely to have a coronal place than another place) and is “triggered” by a dorsal or labial consonant (i.e., the changing consonant acquires a dorsal or labial place). It is more frequently regressive or anticipatory in direction (i.e., an earlier consonant becomes like a later one) than progressive or preservatory. The stereotypical CCH form of *doggy* [gɔgi] (as opposed to [dɔdi]) exhibits all these characteristics.

These patterns can be modeled effectively using autosegmental representations, where contact between the consonants on the consonantal tier allows a coronal consonant underspecified for place to acquire its specification via spreading or copying of a [labial] or [dorsal] feature (Goad, 2001; Stoel-Gammon & Stemberger, 1994). These analytical goals can be met through the use of phonological rules (Smith, 1973) or constraints (Pater & Werle, 2003), which can also specify the direction of the assimilation. While this type of approach achieves high levels of descriptive adequacy, it generally fails to provide a satisfactory answer to why equivalent processes are conspicuously missing from adult phonology. Suggestions that children outgrow early rules (Smith, 1973) or constraints (Pater, 1997), that the domain to which a phonological restriction or process applies changes between childhood and adulthood (Iverson & Wheeler, 1987; Pater & Werle, 2003), or that the drive to copy features diminishes as children grow older (Goad, 2001) all seem to be ad hoc solutions that only raise questions as to why continuity in other phonological generalizations are not disrupted in the same way.

A different approach to CCH focuses on the role of underdeveloped motor control in children. According to the Frame/Content model, for instance, sound patterns that involve repetition of the same articulatory movement, as in CCH, are favored by the developing system because they reduce the complexity of speech production (Davis et al., 2002). In their A-Map model, McAllister Byun et al. (2016) argue that habitual articulatory errors are linked to phonologically expressed reflexes that respond to the demand to produce the most stable form that speakers can manage at the level of their motor development (“Precision,” in their terminology). In young children, Precision can override the drive to produce a form that matches the adult target (“Accuracy”), the result of which can be CCH. In adults, Accuracy usually outranks Precision except in speech errors. This type of approach provides a principled account of why articulatory mechanisms that underline CCH may not affect adult phonology. However, it appears unable to explain why, if CCH reflects forms favored by an underdeveloped articulatory system, adult languages appear to avoid such forms. Forms that are articulatorily easier for children to produce should also be easier for adults to produce and, therefore, usually preferred, not avoided, in phonological systems

While all the analyses discussed above assume that CCH manifests as non-local place assimilation between consonants within a target word, Levelt (1994) and Fikkert and Levelt (2008) contend that CCH, at least initially, is part of a broader assimilatory process involving both consonants and vowels. During the initial developmental phase, the domain for a place specification in children’s lexical representation encompasses the entire word (“one word, one place-of-articulation feature”). The authors support this proposal with examples from Dutch-learning children who produce forms such as [tɪt] for the target /prɪk/ (“injection”). Here, the “harmonized” consonants do not derive the place feature from the consonants in the target, but from the front vowel [ɪ], which shares a coronal feature with [t]. In subsequent developmental stages, apparent occurrences of CCH arise when children overgeneralize frequent patterns of place distribution observed in their linguistic input. For example, the tendency for labial sounds to appear word-initially can lead Dutch children to produce [fup] for /sup/ (“soup”). The notion that early word production reflects unsegmented phonological representation resonates with “whole-word” approaches to child phonology (e.g., Vihman, 2019; Waterson, 1971).

In a different perspective on CCH that also focuses on representational factors, apparent harmony in child forms is seen as a response to memory-related pressure on phonolexical encoding and access. This possibility has been discussed by Hansson (2010) and Vihman (1978), who draw parallels between CCH and adult speech errors, both of which are thought to occur when the speaker encounters difficulties in accurately remembering or recalling sounds in a word. Hansson (2010) proposes that errors in phonological encoding during speech planning explain both CCH and consonant harmony in adult phonology, although the latter is known to occur only between similar consonants sharing major place, such as /s/ and /ʃ/. According to Hansson (2010), the reason encoding errors affect sounds that differ in major place (e.g., /d/ and /g/) in children but not in adults is that children find such sounds more similar to each other than adults do: in a developing system, major place of articulation is less entrenched as a phonological contrast signaling lexical distinctions. Vihman (1978) takes a slightly different approach, seeing CCH as a way of coping with the difficulty in encoding and retrieving dissimilar consonants within the same word. Under this interpretation, CCH alleviates the burdens of representing all sounds in newly acquired words, thereby simplifying the phonological load in the children’s lexicon. Consonant similarity in CCH forms can reduce the amount of phonological information that needs to be encoded, addressing memory constraints and representational challenges. Such challenges may contribute more generally to various discrepancies between the phonological forms produced by children and the adult targets, including phenomena like CCH, metathesis, and segment deletion or omission (Aitchison & Chiat, 1981; Vihman, Ota, Keren-Portnoy, Choo, & Lou, 2023; Vihman, Ota, Keren-Portnoy, Lou, & Choo, 2023). The next section will explore additional arguments and evidence supporting this interpretation of CCH.

### 1.3 The rold of lexical memory constraints

If CCH is at least partially a response to the challenges of encoding and retrieving dissimilar consonants within a word, it likely favors maximal redundancy, manifesting as identical consonantal sounds. Supporting this hypothesis, research indicates that adults more effectively learn novel words with repeated consonants (e.g., /kuka/) than those without such repetition (e.g., /kuna/) (Basnak & Ota, 2024; Ota et al., 2021). In one experiment, Basnak and Ota (2024) familiarized adult English speakers with various novel words as names of unfamiliar objects. All the critical novel words had the structure [consonant-vowel-consonant-vowel] and contained identical consonants (e.g., /kuka/), identical vowels (e.g., /kunu/), or dissimilar consonants and vowels (e.g., /kuna/). Participants displayed the highest recall accuracy for words featuring repeated consonants. This effect may be underpinned by a broader cognitive principle: nearby repeated elements, whether they are letters, musical tones, or visual objects, facilitate adults’ serial recall by allowing these repetitions to be processed as unified elements or “chunks.” This reduces the number of discrete items to remember, effectively compressing the information during encoding (Chekaf et al., 2016; Crowder, 1968; Endress et al., 2007; Feldman, 2000; Henson, 1998; Monaghan & Rowson, 2008).

Moreover, there is evidence that both children and adults may resort to repeating consonants in words when faced with encoding or retrieval difficulties. Aitchison and Chiat (1981) found that children aged 4 to 9 often inaccurately recalled unfamiliar animal names with repeated consonants (e.g., [kuːkuː] for *kudu*, [kəkuːn] for *racoon*, [kaːk] for *yak*, and [kændɪkuː], [gændiguːt], [dændiː] for *bandicoot*). Similarly, in the study by Basnak and Ota (2024), when adult participants made errors in their recall of novel disyllabic words containing different consonants, they produced them with repeated consonants 17% of the time, a rate significantly higher than the 9% chance level. These findings suggest that forms with identical consonants may be recruited as a fallback when encoding or retrieval of a learned form is taxed. This effect may be rooted in a domain-general processing bias on remembering ordered strings. For example, adult participants tasked with serial recall of auditory letters frequently repeat items even in the absence of any repetitions in the input (Conrad, 1965).

The notion that CCH is driven by whole segmental repetitions is not only consistent with evidence from word learning across various age groups and serial recall experiments but also in line with typological generalizations of phonological patterns and core principles of theoretical phonology. As noted earlier, most languages exhibit a dispreference for intravocalic consonants sharing a major place of articulation (e.g., /f. . .p/). Characterizing CCH merely as consonant place harmony, akin to an adult phonological constraint or process, would, therefore, appear to contradict this prevalent tendency. However, the occurrence of identical consonants (e.g., /p. . .p/) often escapes such restrictions on same-place co-occurrence, even though they too, by definition, share a major place of articulation (Gallagher, 2013; MacEachern, 1999). Indeed, in certain languages that typically suppress non-identical place-sharing consonants, words with identical consonants are not only exempt from these restrictions but are also notably overrepresented (Coetzee & Pater, 2008; Graff & Jaeger, 2009).

**Figure 1.**
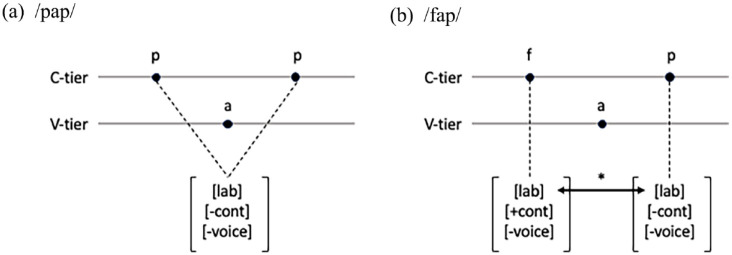
Autosegmental representations of C_1_VC_2_ sequences with (a) identical C_1_ and C_2_ on the consonantal tier and (b) adjacent place-sharing C_1_ and C_2_ (C = consonant, V = vowel).

A formal explanation for this “identity effect” might involve employing the representational tools of autosegmental phonology (Goldsmith, 1976; McCarthy, 1986). In such an analysis, two instances of /p/, as shown in Figure 1(a), could be interpreted as a single feature bundle linked to multiple positions on the consonant tier. If the suppression of place-sharing consonants originates from a phonological constraint against adjacent occurrences of the same major place feature, then the repeated /p/ in Figure 1(a) would escape this constraint because there is only one occurrence of the labial feature. By contrast, the configuration of non-identical labial consonants /f/ and /p/ in Figure 1(b) would violate this constraint because there are two separate labial features adjacent to one another on the consonantal tier. The representation in Figure 1(a) further supports the notion that the identity of the consonants facilitates information compression, offering a memory advantage by simplifying encoding and retrieval.

### 1.4 Prevelance of consonant repetitions in CCH

The discussion above indicates that the aspects of CCH involving full assimilation or consonant repetition might align with empirical findings on memory, word learning, typological patterns, and phonological theory. However, the extent to which CCH can be attributed solely to consonant repetition merits further investigation. Consider the documented examples of CCH produced by a child named Trevor between the ages of 17 and 25 months (Compton & Streeter, 1977), as discussed by Pater and Werle (2003) and presented in Table 1.

**Table 1.** Examples of CCH Produced by Trevor.

Adult target	Child form(s)	Strictly identical consonants	Identical if voice is ignored
*dog*	[ɡoɡ], [ɡoːɡ]	✔	✔
*coat*	[kok]	✔	✔
*cat*	[kæːg]		✔
*tickle*	[ɡiːɡuː]	✔	✔
*bug*	[ɡʌɡ]	✔	✔
*cup*	[kʌk]	✔	✔
*pickle*	[ɡɪɡʊ]	✔	✔
*bed*	[bɛːp]		✔
*butter*	[bʌbə]	✔	✔
*top*	[pap]	✔	✔
*duck*	[ɡʌk]		✔
*take*	[keːk]	✔	✔
*boat*	[bop]		✔

Out of 13 target words, nine instances exhibit forms where the child produced two identical consonants ([k…k], [g…g], [p…p], or [b…b]). That still leaves four cases that do not feature repetitions of the same consonant. However, all those cases have two transcribed consonants that differ only in voice ([k…g], [g…k] or [b…p]). It is known that transcription of children’s voicing contrast in the first 2 or 3 years cannot be reliably interpreted due to large variability in the realization of the phonetic correlates as well as a mismatch in the voiced/voiceless phonetic boundary between children and adult listeners (Macken & Barton, 1980; Scobbie et al., 2000). Furthermore, experimental studies suggest that 2-year-olds may not consistently detect changes in consonant voicing in newly acquired words (e.g., the change from /dibo/ to /tibo/; Quam & Swingley, 2023), pointing to the fragility of voicing encoding during early childhood. Considering these factors, there is a plausible argument that the transcribed differences in voicing might actually reflect the child’s intention to produce identical sounds. If this interpretation is accepted, it could be argued that all examples in Table 1 involve some form of consonant repetition, expanding the relevance of this phonological feature within the framework of CCH analysis.

Another complication arises with the examples presented in Table 1, where all target consonants are stops. This uniformity in manner means that, absent voicing considerations, place assimilation, and segment repetition are indistinguishable in analysis. For example, the production of the word *dog* as [ɡɔɡ] could result either from the target /d/ adopting a dorsal place feature from /ɡ/, or simply from repeating the /ɡ/ for both consonantal positions. The only way to clearly differentiate between these interpretations is to examine target words that vary in both major place and manner of articulation. For instance, if the target word *nap* is produced as [pap], this suggests that segment repetition is more likely than place assimilation. Conversely, if *nap* is rendered [map], this would support the hypothesis of place assimilation over segment repetition.

### 1.5 Purpose of the present study

The discussion above shows that the extent to which CCH comprises identical consonant usage is an empirical matter requiring detailed investigation. Previous quantitative analyses, such as Vihman’s (1978) study, have examined the proportion of CCH instances that involve segment repetition. In her research, Vihman categorized CCH occurrences in 13 children into “total assimilation” (segment repetition) and “partial assimilation” (where the two consonants differed by at least one feature), finding that on average, 63% of cases constituted total assimilation. However, this analysis considered consonants differing only by voice as partial assimilation and did not distinguish between assimilation types that involve only place (e.g., *dog* as [ɡɔɡ]) versus those involving both place and manner (e.g., *nap* as [bap] or [pap]). The aim of the present study is to conduct a similar analysis yet with refined methodology that addresses these distinctions. By doing so, we can more accurately assess the extent to which CCH can be interpreted as segmental repetition.

The analysis reported below was conducted using two distinct datasets of transcribed spontaneous word productions. Unlike many previous studies of CCH, which relied on data from diary-style recordings transcribed “on the fly” by parents or researchers without additional records for verification (e.g., Compton & Streeter, 1977; Smith, 1973), this study utilized only transcripts that were supported by corresponding audio or video recordings to ensure the verifiability of the data. The first dataset included production data from a single recording session with 40 children aged 1 to 2 years, who were speakers of five different languages: English, French, Finnish, Japanese, and Mandarin. The second dataset comprised longitudinal data from seven English-learning children, tracking their language development from 1 to 3 years of age.

Two critical methodological decisions, previously discussed, were applied to both datasets. First, the analysis prioritized target words that contained two supraglottal consonants differing in both place and manner, such as the word *nap*, which features a coronal nasal and a labial stop. These will henceforth be referred to as “NAP-type target words” or simply “NAP targets.” Second, voicing in the transcripts was disregarded, allowing for consonants that share place and manner, such as [k] - [ɡ] and [f] - [v], to be treated as potentially identical. If CCH primarily reflects place assimilation, we would expect most changes in NAP targets to involve only place agreement (e.g., [mæp], [næt], [mæb], or [næd]). Conversely, if CCH is driven by a bias toward maximizing redundancy in memory, then we would predict that most alterations in NAP targets would result in consonants agreeing in both place and manner (e.g., [pæp], [pæb], [bæp], or [bæb]).

## 2 Cross-linguistic data at the “25-word” point

### 2.1 Method

#### 2.1.1 Data

The cross-linguistic data analyzed here are transcripts of spontaneous words produced by 1- to 2-year-olds examined in the study by Vihman, Ota, Keren-Portnoy, Choo, and Lou (2023) and by Vihman, Ota, Keren-Portnoy, Lou, and Choo (2023). These were taken from several previous studies on early production in American English (Vihman & McCune, 1994), British English (DePaolis et al., 2016; Keren-Portnoy et al., 2010), Finnish (Kunnari, 2000; Savinainen-Makkonen, 2001), French (de Boysson-Bardies et al., 1992; Wauquier & Yamaguchi, 2013), Japanese (de Boysson-Bardies et al., 1992; Ota, 2003), and Mandarin (Lou, 2021). Data from these studies comprise audio- or video-recorded naturalistic speech productions of the target children, transcribed by phonetically-trained native speakers of each language. Transcription reliability information for each data source is given in Table 2.

**Table 2.** Data Sources and Transcription Reliability.

Source	Language (number of children included in the current study)	Transcription and reliability
Vihman and McCune (1994)	American English (6)	Delayed intra-transcriber agreement for 1 transcriber on place and manner of consonants: 81.7%–83.6%^a^
Keren-Portnoy et al. (2010)	British English (1)	Inter-transcriber agreement among 3 transcribers on place and manner of consonants: 80%–86%
DePaolis et al. (2016)	British English (8)	Inter-transcriber agreement among 3 transcribers (reported only for consonantal productions in the prelinguistic 10-month recordings): 65%–72%
Kunnari (2000)	Finnish (4)	Inter-transcriber agreement between 2 transcribers on place and manner of consonants: 87.7%
Savinainen-Makkonen (2001)	Finnish (1)	One transcriber. No reliability checks performed.
de Boysson-Bardies et al. (1992)	French (5), Japanese (5)	Inter-transcriber agreement between 2 transcribers on place and manner of consonants: 80% (French). 80%–89% (Japanese)
Wauquier and Yamaguchi (2013)	French (3)	Two transcribers for different portions of data. No reliability checks performed.
Ota (2003)	Japanese (3)	One transcriber. No reliability checks performed.^b^
Lou (2021)	Mandarin (5)	One transcriber. No reliability checks performed on segments.

a Audio-recordings available at https://phon.talkbank.org/access/Eng-NA/StanfordEnglish.html.

b Audio-recordings available at https://phon.talkbank.org/access/Japanese/Ota.html.

From these sources, Vihman, Ota, Keren-Portnoy, Choo, and Lou (2023) and Vihman, Ota, Keren-Portnoy, Lou, and Choo (2023) extracted all child forms and their adult targets from the first half-hour recording session during which each child spontaneously produced at least 25 lexical items by type count. Children who produce 25 or more word types in a half-hour session have been found to have about 50 words in their productive vocabulary (Vihman & Miller, 1988). Although this means the children sampled differed in their chronological age, they were at similar points of development in terms of their lexical advance. The number and age of children and the number of spontaneously produced words are summarized in Table 3. Further details of this dataset can be found in the study by Vihman, Ota, Keren-Portnoy, Lou, and Choo (2023).

**Table 3.** Number of Children, Age, and Number of Words in the Data Sources: Cross-linguistic Data.

Language	Number of children	Child age in years and months: mean (range)	Number of word types per child: mean (range)
American English	6	1;4 (1;4–1;5)	35.8 (29–45)
British English	9	1;6 (1;3–1;9)	38.7 (27–61)
Finnish	5	1;6 (1;3–1;10)	34.2 (25–53)
French	8	1;6 (1;3–1;8)	32.5 (27–46)
Japanese	7	1;9 (1;3–2;3)	43.1 (26–72)
Mandarin	5	1;5 (1;5–1;6)	37.6 (34–43)

#### 2.1.2 Inclusion criteria for target words

The current study focused on a subset of this dataset, filtered through the following criteria. First, the target word had to be of the structure C_1_VC_2_ V(C), where C was a consonant (including a geminate or long consonant), V was a vowel, and (C) was an optional consonant. This was done to allow cross-linguistic comparison, as C_1_VC_2_ V(C) is a structure that is licensed in all the five languages examined here. Second, the first and second consonants in the target had to be supraglottal consonants that differ both in major place and manner (i.e., a NAP target). This filtering process was carried out manually by this author. Table 4 shows the number of items that met these criteria for each language and some examples.

**Table 4.** Number of C_1_VC_2_ V(C) NAP Target Words and Examples: Cross-linguistic Data.

Language	Number of items	Examples
English	21	/bʌni/ *bunny*, /kɑfi/ *coffee*, /ʤəræf/ *giraffe*, /bəlun/ *balloon*
Finnish	39	/kɑlɑ/ ‘fish’, /pɑlːo/ ‘ball’, /nuke/ ‘doll’, /vetːæ/ ‘water’
French	35	/kanaʁ/ ‘duck’, /ʃapo/ ‘hat’, /lapɛ̃/ ‘rabbit’, /balɔ̃/ ‘balloon’
Japanese	24	/bo:ɕi/ ‘hat’, /kame/ ‘turtle’, /kɯʦɯ/ ‘shoes’, /neko/ ‘cat’
Mandarin	13	/fei1ʨi1/ ‘airplane’, /lau2xu3/ ‘tiger’, /ʦui3pa1/ ‘mouth’, /ɕi1kua1/ ‘watermelon’

*Note.* Target words are shown in phonetic transcription followed by a gloss, except for English words, where the orthographic form is given instead of a gloss. Numbers on Mandarin syllables indicate tone (1 = level, 2 = rising, 3 = fall-rise, 4 = falling).

#### 2.1.3 Classification of non-target-like consonant production in child forms

The transcription of each form produced by the child was classified into one of the following categories based on how C_1_ and C_2_ in the C_1_VC_2_ V(C) target were realized in the child form.

1. Segmental: There is a change in C_1_ or C_2_ that is attributable to a common segmental process independent of the other consonant(s). For example, the realization of the target flap /ɾ/ in Japanese as [d] or [n] occurred across several words and was judged to be a case of fortification of the flap (e.g., /kiːɾo/ ↦ [kiːdo] ‘yellow’; /ɾapːa/ ↦ [dapːa] ‘horn’; /ɾaiõɴ/ ↦ [naːjoɴ] “lion”). Production of a target retroflex affricate as an alveolar stop in Mandarin was also considered a case of segmental process (e.g., /ʈʂɤ4kɤ4/ ↦ [tiao4kˤɤ4] or [tɤ1kɤ4] ‘here’; /ʈʂʰɤ1ʈʂʰɤ1/ ↦ [tɤ1tɤ4] ‘car’; /ʈʂuo1tsi0/ ↦ [tɤ1ʈʂai4] “table”).
2. Agreement—place only: C_1_ and C_2_ in the child form share the same major place of articulation but not the manner of articulation. For example, the consonants corresponding to C_1_ and C_2_ in the child form [wɑ:mɪ] for *water* both have a labial place, but not the same manner.
3. Agreement—manner only: C_1_ and C_2_ in the child form share the same manner of articulation, but not place in the child form. Examples include /kɑlɑ/ ↦ [kɑtɑ] “fish (Finnish)” and /banan/ ↦ [baɖa] “banana (French).”
4. Agreement—place & manner: C_1_ and C_2_ in the child form share the same place and manner of articulation. Because we are disregarding voice for the purpose of this analysis, this includes words with any pair of C_1_ and C_2_ sharing a major place and manner even if they have different voicing. Examples include /kɑfi/ ↦ [gɑːgi] *coffee* (English), /ʃapo/ ↦ [papoː] “hat (French),” and /ɾapːa/ ↦ [bapːa] “horn (Japanese).”
5. Deletion—C_1_: A consonant corresponding to C_1_ is absent from the child form. Examples include /nɑpːi/ ↦ [ɑpːi] “button (Finnish)” and /kase/ ↦ [aʧe] “broken (French).”
6. Deletion—C_2_: A consonant corresponding to C_2_ is absent from the child form. Examples include /kynæ/ ↦ [ku.a] “pen (Finnish)” and /pɑlːo/ ↦ [pɑː.o] “ball (Finnish).”

## 2.2 Results

**Figure 2.**
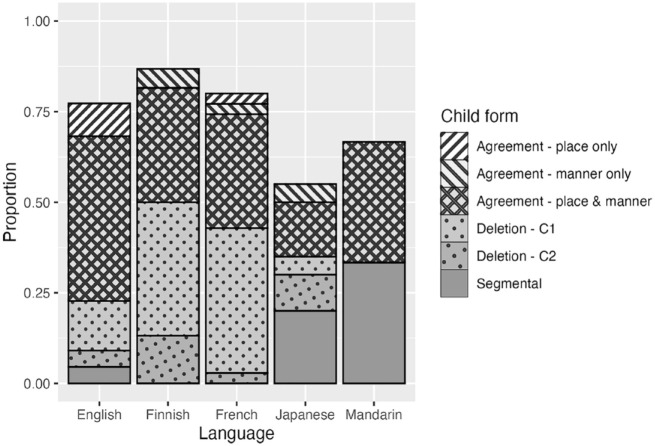
Non-target-like child form types produced for C_1_VC_2_ V(C) NAP targets: Cross-linguistic data.

Figure 2 displays the proportion of the six types of child form produced for all C_1_VC_2_ V(C) targets in which C_1_ and C_2_ differed in both place and manner. Before we discuss the details of the agreement patterns between C_1_ and C_2_, it is worth commenting on three cross-linguistic differences observable in Figure 2. First, a large proportion of cases fall under the category “Segmental” in Mandarin. As mentioned in the Method section, this is mostly due to a common substitution process whereby a target retroflex affricate is realized as an alveolar stop (e.g., /ʈʂɤ4kɤ4/ ↦ [tɤ1kɤ4] “here”). The “Segmental” cases in the Japanese data were accounted for by a common process in that language that substitutes [d] or [n] for /ɾ/ (e.g., /kiːɾo/ ↦ [kiːdo] “yellow”). Second, Finnish and French exhibit a large proportion of words with the first consonant deleted from the child form. This is a pattern repeatedly observed in previous work on these languages and has been attributed to the perceptual salience of word-medial geminates in Finnish and the phrase-final accent in French, which arguably distract the learner’s attention away from the initial consonant (Savinainen-Makkonen, 2007; Vihman et al., 2004; Vihman & Majorano, 2017). Third, the overall proportion of words that undergo an assimilatory process is lower in Japanese and Mandarin than in English, Finnish, and French. This cross-linguistic difference has been discussed by Vihman, Ota, Keren-Portnoy, Choo, and Lou (2023) and by Vihman, Ota, Keren-Portnoy, Lou, and Choo (2023), who suggest that a small syllable inventory (such as those in Japanese and Mandarin) allows learners to retain word forms by recombining remembered syllables and successfully produce “variegated” words (i.e., word forms containing two different consonants) without assimilating the consonants. I will return to this point in the General Discussion.

**Figure 3.**
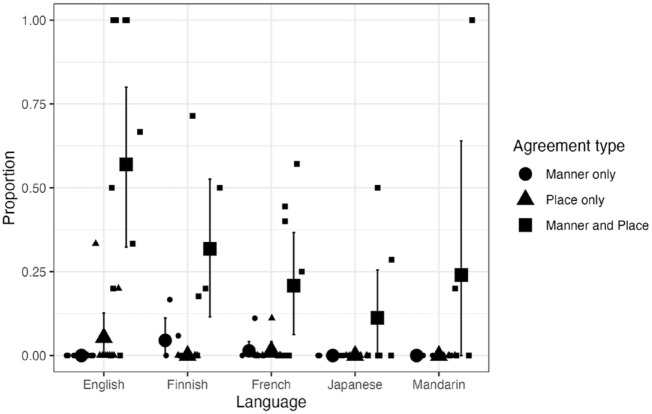
Agreement types produced for C_1_VC_2_ V(C) NAP targets: Cross-linguistic data.

Let us now turn to the output types that are critical to our investigation, the agreement categories. If CCH is induced by assimilation of major place, we expect to see more NAP targets produced with consonants sharing place (“Agreement—place only”) than with consonants sharing both place and manner (“Agreement—place & manner”). In contrast, if CCH is driven by lexical memory reduction, we expect to see a bias toward segment repetition. We therefore predict more place-and-manner agreement than place-only agreement in children’s production of NAP targets. The agreement types in Figure 2 are re-plotted in Figure 3, along with the distribution of individual means. Although there were considerable individual differences, the group mean proportion of child forms with place-and-manner agreement was higher than both the mean proportion with place-only agreement and that with manner-only agreement in all five languages.

To statistically examine whether child forms of NAP targets were more likely to have place-and-manner agreement than other agreement types, a Bayesian, mixed-effects, multinomial logistic regression model was applied to the data using the brms package (Bürkner, 2021) in R (R Core Team, 2024). Child forms of NAP targets (132 observations) were classified into four categories: place-and-manner agreement, place-only agreement, manner-only agreement, and “other” (any other forms, including those that were adultlike). The model included Language as a fixed effect and random intercepts for children and was specified as Child form ~ 1 + Language + (1 – Child). The reference level was set to place-and-manner agreement for Child form and English for Language. Priors were set using a Student *t* distribution with 3 degrees of freedom, a mean of 0, and a scale parameter of 2.5.

The results indicated evidence for differences in the intercepts and some agreement-language effects. The model showed that, for the reference language (English), child forms were less likely to have place-only agreement (β = −2.36, 95% CI [−5.83, −0.42]) than to have place-and-manner agreement. It also yielded results suggesting less manner-only agreement (β = −67.10, 95% CI [−399.46, −3.50]) than place-and-manner agreement, although this estimate may be unreliable because of a low bulk effective sample size (270). The model also indicated that Finnish had even less place-only agreement than place-and-manner agreement (β = −39.29, 95% CI [−284.11, −0.37]). There was also an interaction involving Finnish (vs. English) and manner-only agreement (vs. place-and-manner), but one that counteracts the effect found in English (β = 64.75, 95% CI [1.06, 395.33]), suggesting a lack of reliable difference between place-and-manner and manner-only in Finnish. Japanese had more child forms in the “other” category relative to English (β = 2.98, 95% CI [0.72, 5.86]). There were no other language-related statistical differences. Thus, for English and Finnish, the proportion of place-and-manner agreement was statistically higher than that of place-only agreement. There was no evidence that the other three languages differed from English and Finnish in this respect. There was also some evidence that there was more place-and-manner agreement than manner-only agreement in English, and the only language that differed from English in this regard was Finnish, which had a reduced difference between those two types of agreement. Overall, the quantitative analysis is largely consistent with the prediction that place-and-manner agreement is more prevalent than place-only agreement and manner-only agreement.

Table 5 lists some examples of child forms with place-and-manner agreement produced for NAP targets. Table 6 gives all cases of place-only agreement (English and French) and manner-only agreement (Finnish and French) found in the data. Japanese and Mandarin had no instances of place-only or manner-only agreement.

**Table 5.** Examples of Place-and-Manner Agreement: Cross-linguistic Data.

Language	Target	Child form	Gloss	Child (age)
English (UK)	sɜːkəl	geːgə	*circle*	Lewis (1;5)
English (US)	bəluːn	bɛbɛ	*balloon*	Timmy (1;5)
English (UK)	ræbɪt	bæbɪtʰ	*rabbit*	Flora (1;7)
Finnish	kɑlɑ	ʎɑʎɑ	fish	Mira (1;5)
Finnish	lopːu	popːu	finish	Sini (1;3)
Finnish	ʋetːæ	titːæ	water	Eliisa (1;3)
French	ʃapo	bobo	hat	Laurent (1;5)
French	divɑɑ̃	vəvə	couch	Basile (1;3)
French	lapɛ̃̃	paːpa	rabbit	Carole (1;3)
Japanese	baʨ:i	babɪ	dirty	Kenji (1;7)
Japanese	gʸɯːɲʸɯː	diːdi	milk	Kenta (2;3)
Japanese	ɾapːa	bap:a	horn	Kenta (2;3)
Mandarin	ɕi1kua1	pˤɤ1pˤɤ1	watermelon	Shi (1;5)
Mandarin	ʈʂɤ4kɤ0	ku4ku0	this	Xinyu (1;5)

In Tables 5 and 6, targets and child forms are presented in phonetic symbols. For English, orthographic forms are given instead of glosses.

**Table 6.** Examples of Place-Only and Manner-Only Agreement: Cross-linguistic Data.

Agreement	Language	Target	Child form	Gloss	Child (age)
Place only	English (UK)	ʤɪrɑf	puwæɸ	*giraffe*	Flora (1;7)
Place only	English (US)	wɑt̬ɚ	wɑ:mɪ	*water*	Emily (1;4)
Place only	French	lamā	pæmā	the hand	Noël (1;6)
Manner only	Finnish	lopːu	topːu	finish	Sini (1;3)
Manner only	Finnish	kɑlɑ	kɑtɑ	fish	Mira (1;5)
Manner only	French	banan	baɖa	banana	Noël (1;6)

### 2.3 Discussion

The agreement pattern occurring between consonants in this dataset is quite clear across the five languages. In the vast majority of the cases, we find the two consonants in the child form to be identical (disregarding voicing). If CCH is indeed a harmony process of major place, we would expect to see more cases where the manner of the target consonant is retained when its place assimilates with another consonant in the form produced, or at least we would expect to see more variation in the manner of the consonant whose place is “assimilated” to the other consonant. These predictions are not supported. When the two consonants in the child form agree in place, they also strongly tend to agree in manner. Overall, then, the cross-linguistic analysis presented here is more consistent with the view that the process that underlies CCH is one that repeats the same consonant rather than one that assimilates consonants in place.

There are some issues and limitations with this analysis, however. First, the use of the 25-word criterion meant that the samples came only from a relatively early stage of word production (the mean age for each language group was between 1;4 and 1;8). Some examples of place-only agreement for NAP targets previously reported in the literature are from 2-year-olds (e.g., *nipple* ↦ [mibu]/[mipu]/[mib̥u] and *some* ↦ [fʌm]; Smith, 1973), suggesting that such a process may be more prevalent in words produced by slightly older children. Second, to ensure cross-linguistic comparison, the analysis here was limited to CVCV(C) targets. But as exemplified in those listed in Table 1, many examples of CCH cited in the literature are for CVC targets (e.g., [gɔg] *dog*, [gʌk] *duck*). This leaves the possibility that the results of the analysis here are specific to CVCV(C) targets. Third, one might argue that these results are partly an artifact of the phonemic inventories of the languages studied. In all five languages, dorsals are constrained in manner, and liquids are constrained in place. Dorsal consonants can be plosive (/k, g/) or nasal (/ŋ/), but a dorsal nasal (/ŋ/) cannot be in a syllable onset (except as an intervocalic allophone of /g/ in some Japanese dialects). Mandarin also has a fricative whose realization varies between a dorsal [x] and glottal [h]. But otherwise, there are no other dorsal phonemes in these languages. A liquid is always coronal except as a positional allophone of /l/ in some variants of English where it can be dorsal (or “dark”). If children tend to produce only sounds that are phonemically licit in the language or if transcribers tend to ignore non-phonemic differences in children’s word production, words containing one of these sounds will be constrained in the range of place-only agreement they can accommodate. For example, a target word such as *Mickey* can be produced with either one of the consonants repeated ([mɪmi], [kɪki]) but with only one type of place-only assimilation, in which the stop in C_2_ adopts the labial place of C_1_ ([mɪpi]), because the other possibility—that is, the nasal in C_1_ adopting the dorsal place of C_2_—results in a form that is phonotactically impossible in English (i.e., [ŋɪki]). As a result, the analytical approach adopted here may be biased against place-only agreement. An obvious way to avoid this problem is to analyze only those targets (e.g., *nap*) that could be equally plausible in being produced with place-and-manner agreement (e.g., /pæp/, /næn/) or place-only agreement (e.g., /mæp/, /næt/). For such an analysis, we need a larger set of data than the current one which contained only 132 observations of NAP-word productions across five languages.

To address these issues, a similar analysis was applied to longitudinal data from English children covering a 2-year period between 1;0 and 3;0. Both CVC and CVCV(C) targets were examined in seven children. Altogether, the data contained over 26,000 tokens of NAP targets, which allowed for the type of analysis suggested above. The results are reported in the next section.

## 3 Longitudinal data from English-learning children

### 3.1 Method

#### 3.1.1 Data

The data analyzed in this section were transcripts of words spontaneously produced by seven children learning North American English: two (Julia and Sonya) from the Goad corpus (Parsons, 2006) and the remaining five (Alex, Lily, Naima, Violet, and William) from the Providence corpus (Demuth et al., 2006). The children in the Goad corpus were from Montreal, Canada, and those in the Providence corpus were from southern New England in the United States. Data from a sixth child in the Providence Corpus were not included in the analysis as he was later diagnosed with Asperger Syndrome. All seven children were audio-recorded (and in the case of the Providence children, also video-recorded) approximately every other week, and their productions were phonetically transcribed. Data in the Goad corpus were triple-blind transcribed and validated by two of the transcribers, who selected the best transcription based on further inspection of the recording whenever there was a disagreement. Reliability was checked only for voicing of plosives, which was the focus of the analysis. The three transcribers agreed 94.06% of the time. Data in the Providence corpus were transcribed by one person. A second transcriber then transcribed 10% of the data from each recording to assess reliability. Agreement between the two transcribers averaged 84%. Both digitized corpora were accessed through PhonBank (Rose & MacWhinney, 2014). For the purpose of the current analysis, only transcriptions of recordings made between ages 1;0 and 3;0 were analyzed.

#### 3.1.2 Inclusion criteria for target words

Two criteria were used to select the target words for the initial phase of the analysis. First, the target words had to have either one of the two structures: C_1_VC_2_ (e.g., *ball, cat*), or C_1_VC_2_ V(C) (e.g., *doggie, rabbit*), where C = consonant and V = vowel. Second, C_1_ and C_2_ in the target word had to be supraglottal consonants that differ in major place of articulation and manner of articulation (NAP targets). Further exclusion applied to the second analysis is explained later. Data extraction from the corpora was carried out using phonex regular expressions on Phon (Hedlund & Rose, 2020). The number of cases analyzed is summarized in Table 7.

**Table 7.** Number of NAP Targets Analyzed: Longitudinal English Data.

Child	CVC		CVCV(C)	
	Type	Token	Type	Token
Alex	105	2,299	59	289
Julia	99	1,034	78	497
Lily	181	4,784	154	1,452
Naima	218	8,747	229	3,084
Sonya	70	664	53	310
Violet	73	645	70	488
William	124	1,988	73	833

#### 3.1.3 Classification of non-target-like consonant production in child forms

The transcription of each word form produced by the child was examined, and non-target-like realizations of C_1_ and C_2_ in C_1_VC_2_ and C_1_VC_2_ V(C) words were classified into one of the same categories used in the cross-linguistic analysis. The operationalizations are repeated below, along with relevant examples from the corpora. This step was carried out manually by this author.

1. Segmental: There is a change in C_1_ or C_2_ that is attributable to a common segmental process independent of the other consonant(s). The processes observed include fortification of fricatives/affricates (e.g., *some* ↦ [tʌm], Sonja 1;10.7; *chicken* ↦ [tɪkɛn], Naima 2;1.16), vocalization of liquids (e.g., *ball* ↦ [bow], Julia 1;8.10; *rock* ↦ [wɑk], Naima 2;5;18), velar fronting (e.g., *car* ↦ [tɔr], William 1;9.5), and interdental fronting (e.g., *thing* ↦ [fɪŋ], Lily 2;6.4).
2. Agreement—place only: C_1_ and C_2_ in the child form share the same major place of articulation but not the manner of articulation. Examples include *come* ↦ [pʰʌm] (Julia 2;6.5), *tummy* ↦ [tʌni] (Lily 2;2.15), and *bath* ↦ [dæθ] (William 1;10.12).
3. Agreement—manner only: C_1_ and C_2_ in the child form share the same manner of articulation, but not place in the child form. Examples include *lamb* ↦ [næm] (Julia 2;11.15), *Mickey* ↦ [bɪki] (Violet 1;11.28), and *ding* ↦ [niŋ] (William 1;9.12).
4. Agreement—place & manner: C_1_ and C_2_ in the child form share the same place and manner of articulation. Examples include *TV* ↦ [titi] (Lily 1;8.3), *balloon* ↦ [bubu] (Alex 2;1.2), *paws* ↦ [pʰɑp] (Julia 2;11.22), and *licking* ↦ [ɡikɪŋ] (William 1;8.2).
5. Deletion—C_1_: A consonant corresponding to C_1_ is absent from the child form. Examples include *look* ↦ [ʊk˺] (Sonya 2;10.7), *Micky* ↦ [ɪki] (Violet 2;0.27), and *rock* ↦ [ɑk] (William 2;6.26).
6. Deletion—C_2_: A consonant corresponding to C_2_ is absent from the child form. Examples include *knife* ↦ [naj] (Sonya 2;8.12), *sock* ↦ [sɑ] (Alex 2;11.8), and *giving* ↦ [gɪŋ] (Naima 2;0.12). When the transcription showed a glottal stop in place of a supraglottal consonant, it was treated as deletion (e.g., *bunny* ↦ [bʌʔi], *catch* ↦ [kæʔ])

For the descriptive statistics reported below, category assignment was aggregated by every word type per child in four age bins: 1;0–1;5, 1;6–1;11, 2;0–2;5 and 2;6–3;0. For example, between 2;6 and 3;0, Violet targeted the word *sheep* seven times and produced it as [ʃip], [sip], [si], [ti], [ti], [tsi], and [siʃ]. For this age bin, Violet’s productions of *sheep* were classified as 4/7 C_2_ deletion, 3/7 segmental process, and 1/7 place and manner agreement. Note here that tokens showing both deletion and segmental processes (e.g., [ti]) were counted into both proportions. However, when a token exhibited potential segmental process as well as agreement (*wall* ↦ [wɔw]), it was not counted as a case of agreement to avoid overinterpretation of assimilatory processes.

### 3.2 Results

#### 3.2.1 Analysis of overall data

**Figure 4.**
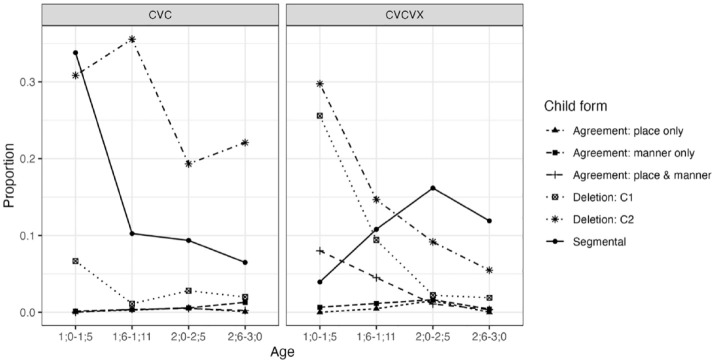
Non-target-like child form types produced for NAP targets: Longitudinal English data.

Figure 4 summarizes non-target-like productions of consonants in all NAP targets. For both C_1_VC_2_ and C_1_VC_2_ V(C) targets, the most prevalent patterns overall were deletion and segmental changes, although the relative proportions between these categories and their developmental trajectories were different between the two target types. There was a higher proportion of C_2_ deletion in CVC targets than in CVCV(C) targets. Conversely, there was a higher proportion of C_1_ deletion in CVCV(C) targets than in CVC targets. The proportion of child forms with non-target-like agreement (i.e., assimilation) was low, particularly for CVC targets.

**Figure 5.**
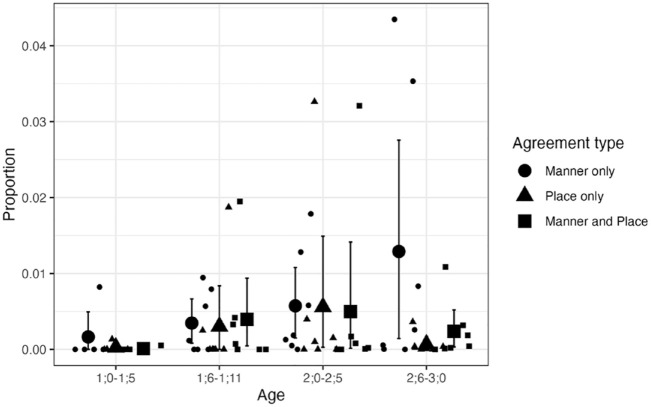
Agreement types for CVC NAP targets: Longitudinal English data. *Note.* Small circles, triangles, and squares show individual means. Large circles, triangles, and squares show group means. Error bars are bootstrapped 95% confidence intervals.

**Figure 6.**
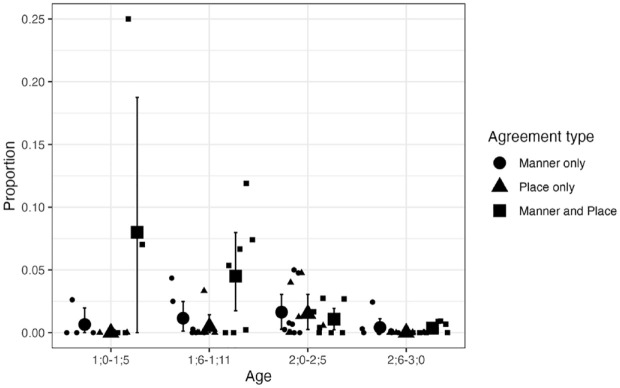
Agreement types for CVCV(C) NAP targets: Longitudinal English data. *Note.* Small circles, triangles, and squares show individual means. Large circles, triangles, and squares show group means. Error bars are bootstrapped 95% confidence intervals.

As our main interest is in the proportions of agreement types, these are re-plotted below but separately for CVC and CVCV(C) targets in Figures 5 and 6, respectively, as the overall levels differ quite dramatically. Figure 5 shows that the proportions of child productions displaying these agreement patterns are very low for CVC targets, with the majority of individual means below 1% at any age range. As can be seen in Figure 6, the proportions are generally higher for CVCV(C) targets, especially between 1;0 and 1;11.

The data were subjected to a Bayesian, mixed-effects, multinomial logistic regression analysis, following the same strategy used for the cross-linguistic data. Separate models were built for CVC and CVCV(C) targets. Child forms of NAP targets (19,540 observations for CVC, and 6,647 observations for CVCV(C)) were classified into four categories: place-and-manner agreement, place-only agreement, manner-only agreement, and “other” (any other forms, including those that were adultlike). The model included Age as a fixed effect, binned to the same four ranges used in the descriptive statistics above, and random intercepts for children and target word and was specified as Child form ~ 1 + Age + (1 – Child) + (1 – Word). The reference level was set to place-and-manner agreement for Child form and 1;0–1;5 for Age. Priors were set using a Student *t* distribution with 3 degrees of freedom, a mean of 0, and a scale parameter of 2.5. Estimates are reported as log odds.

The results for CVC targets produced only one reliable effect. The model showed that child forms for these targets were more likely to fall under the “other” category than place-and-manner agreement (β = 6.40, 95% CI [0.99, 4.65]). No differences emerged between place-and-manner and place-only or between place-and-manner and manner-only. There was no statistical evidence that these relationships differed between the four age bins. These results show that, for CVC NAP targets, each of the agreement types occurred less frequently than target-like productions or productions with deletion or segmental processes. But there was no indication that place-and-manner agreement was more likely than place-only agreement in the child forms.

The results for CVCV(C) targets, in contrast, revealed several differences in the intercepts for Child form and some age-dependent effects. The model suggested that, for the reference age (1;0–1;5), child forms were less likely to have place-only agreement (β = −4.56, 95% CI [−8.45, −1.16]) or manner-only agreement (β = −3.00, 95% CI [−5.17, −1.19]) than place-and-manner agreement. The model also indicated that, relative to the level in the first age bin, the proportion of “other” was higher for 1;6–1;11 (β = 2.73, 95% CI [1.87, 3.65]), 2;0–2;5 (β = 4.62, 95% CI [3.56, 5.79]), and 2;6–3;0 (β = 5.12, 95% CI [3.95, 6.37]). These posterior estimates provide evidence that the relative proportion of place-and-manner agreement decreased in relation to the “other” category between 1;0–1;5 and 1;6–1;11, and also between 1;6–1;11 and 2;6–3;0. The descriptive data summarized in Figure 6 also suggest that the tendency toward place-and-manner over place-only and manner-only disappears after 2;0, but this was not statistically confirmed.

Some examples of child forms with place-and-manner agreement for NAP targets are given in Table 8. For comparison, examples of child forms with place-only agreement are listed in Table 9.

**Table 8.** Examples of Child Forms With Place-and-Manner Agreement: Longitudinal English Data.

Target structure	Target (orthography)	Target (phonetic)	Child form	Child (year;month.day)
CVC	Matt	mæt	dætʰ	Lily (1;8.3)
CVC	one	wʌn	nʌn	William (2;7.8)
CVC	sheep	ʃip	siʃ	Violet (2;8.7)
CVC	pull	pʊl	pub	Alex (2;1.2)
CVC	more	mɔɹ	mɔm	Julia (2;3.20)
CVCV(C)	rabbit	ɹæbɪt	bæbæ	Naima (1;1.11)
CVCV(C)	TV	tivi	titi	Lily (1;8.27)
CVCV(C)	kitchen	kʰɪʧən	ʧɪʧn̩	Julia (2;3.20)
CVCV(C)	balloon	bəlun	bubu	Alex (1;9.22)
CVCV(C)	water	wɑtəɹ	wʌwæ	Naima (1;1.24)

**Table 9.** Examples of Child Forms With Place-Only Agreement: Longitudinal English Data.

Target structure	Target (orthography)	Target (phonetic)	Child form	Child (year;month.day)
CVC	pen	pɛn	dɛn	Violet (2;2.1)
CVC	lamb	læm	bæm	William (1;10.12)
CVC	come	kʌm	pʰʌm	Julia (2;6.5)
CVC	ding	dɪŋ	gɪŋ	Naima (1;5.2)
CVC	look	lʊk	ləd	Lily (3;0.3)
CVCV(C)	Thomas	tɑməs	tɑnɪs	William (2;5.29)
CVCV(C)	camel	kæməl	pʰamʊ	Julia (1;4.25)
CVCV(C)	rabbit	ɹæbɪt	bæwʊt	Violet (2;5.15)
CVCV(C)	tummy	tʌmi	tʌni	Lily (12;2.15)
CVCV(C)	bunny	bʌni	bɑmɛ	William (2;0.28)

#### 3.2.2 Analysis of data without targets containing dorsals and liquids

As previously discussed, an analysis comparing place-and-manner agreement and place-only agreement using all words may underestimate potential place-only agreement in child forms because a dorsal sound in English can only be a stop or a nasal, the latter of which cannot be a syllable onset, and a liquid sound in English can only be coronal. To address this issue, the same analysis was repeated on the subset of the data that excluded target words containing dorsals or liquids. After the exclusion of these words, the subset of data consisted of 9,850 observations of CVC targets (50.4% of all CVC NAP targets) and 3,022 observations of CVCV(C) targets (45.1% of all CVCV(C) NAP targets).

**Figure 7.**
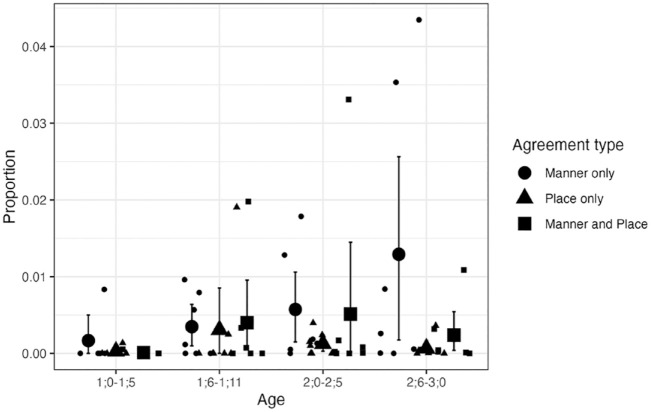
Agreement types for CVC NAP targets excluding words with dorsals or liquids: Longitudinal English data. *Note.* Small circles, triangles, and squares show individual means. Large circles, triangles, and squares show group means. Error bars are bootstrapped 95% confidence intervals.

**Figure 8.**
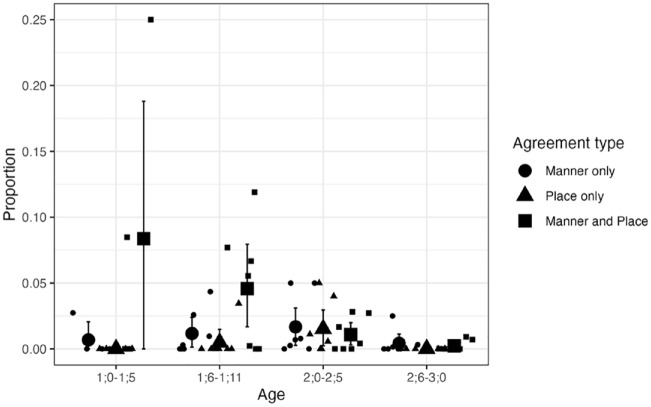
Agreement types for CVCV(C) NAP targets excluding words with dorsals or liquids: Longitudinal English data. *Note.* Small circles, triangles, and squares show individual means. Large circles, triangles, and squares show group means. Error bars are bootstrapped 95% confidence intervals.

The proportions of child forms for these target words that featured place-only, manner-only, or place-and-manner agreement are given in Figures 7 and 8. Overall, the distributions appear similar to those based on the full data.

A Bayesian, mixed-effects, multinomial logistic regression analysis was carried out separately for the CVC and CVCV(C) targets, using the same model specifications and settings of parameters applied to the analysis of the full data. For CVC targets, the model suggested that the children produced more forms under the “other” category than with place-and-manner agreement (β = 5.96, 95% CI [3.52, 9.26]). It also produced posterior estimates indicating that child forms were less likely to have place-only agreement than place-and-manner agreement (β = −106.81, 95% CI [−403.74, −3.47]). However, this outcome may not be reliable as it was based on a tail effective sample size that was too low (310). The models also suggested that the proportion of place-only agreement increased from 1;0–1;5 to 1;6–1;11 (β = 105.81, 95% CI [2.44, 402.42]), 2;0–2;5 (β = 106.95, 95% CI [3.30, 404.14]), and 2;6–3;0 (β = 106.56, 95% CI [3.02, 403.43]), but these results too must be interpreted with caution due to low tail effective sample sizes (310, 309, 310, respectively).

The results for CVCV(C) targets revealed several differences in the intercepts for Child form and some category-age combinations. The model suggested that, for the reference age (1;0–1;5), child forms were less likely to have place-only agreement (β = −4.08, 95% CI [−8.72, −0.03]) or manner-only agreement (β = −4.45, 95% CI [−8.43, −1.51]) than place-and-manner agreement. The model also indicated that, relative to the level in the first age bin, the proportion of “other” was higher for 1;6–1;11 (β = 3.18, 95% CI [0.47, 6.73]), 2;0–2;5 (β = 3.69, 95% CI [0.39, 7.62]), and 2;6–3;0 (β = 4.86, 95% CI [0.85, 3.38]). This reflects an overall decline in the proportion of child forms with agreement between consonants. Unlike the analysis based on overall data, the subset data yielded evidence that the proportion of place-only agreement in child forms was higher in 2;0–2;5 than in 1;0–1;5, (β = 3.92, 95% CI [0.08, 8.10]). The regression coefficients and descriptive data suggest that place-and-manner agreement is not higher than place-only agreement for this period. In general, even when we focus on words that could in theory be produced with place-only agreement within the inventory of English phonemes, place-and-manner agreement was more likely than manner-only and place-only agreement for CVCV(C) targets before 2;0.

### 3.3 Discussion

In sum, the analysis of the longitudinal English data here mostly aligns with the results of the cross-linguistic data as far as the CVCV(C) NAP target words are concerned. Here too, we find that child forms with some form of agreement between C_1_ and C_2_ are more likely to have agreement in both place and manner than in place only. If we disregard voicing, this means the two consonants tend to be identical when there is some type of agreement. This effect was also confirmed when we removed target words containing a dorsal or liquid sound to minimize any bias against place-only agreement. This pattern was most noticeable before the age of 2 years, partly because the overall proportion of child forms with some type of agreement is higher during that period, and there is some indication that the difference between place-and-manner and place-only agreement decreases in the third year.

For CVC NAP targets, however, we did not find robust evidence that agreement in child forms tended to be for both place and manner. The analysis of the overall data yielded no support for the prediction, and while the analysis of the subset data without dorsals and liquids produced results in the predicted direction, there are statistical reasons to be cautious of the interpretation of the outcomes.

## 4 General discussion

### 4.1 Key findings

The aim of this study was to explore the hypothesis that CCH is driven by the cognitive demands children encounter when encoding and retrieving distinct consonants within word forms. The advantage of this perspective is that it addresses the long-standing challenge of providing an account of CCH that is not only descriptively adequate but also consistent in principle with general patterns in later word learning and phonological systems. According to this proposal, CCH is predicted to favor segment repetition, corresponding to place-and-manner harmony, more than place-only assimilation. To distinguish between place-and-manner assimilation and place-only assimilation, we tested this prediction against spontaneously produced child speech for target words featuring consonants that differ in both major place and manner (i.e., NAP targets).

In the analysis using the 25-word point data from five languages, the overwhelming majority of assimilated forms in CVCV(C) target words displayed both place and manner agreement. Likewise, longitudinal data from seven English-learning children, aged 1;0–3;0, generally showed a preference for consonant agreement involving both place and manner over mere place agreement in CVCV(C) targets, although the prevalence of place-and-manner agreement appeared to decrease with age. However, the analyses of CVC targets from the same dataset did not produce definitive results supporting a greater tendency for place-and-manner agreement over place-only agreement. Overall, the evidence suggests that CCH in NAP targets before 2;0 predominantly involves segment repetition in CVCV(C) word structures, but the effect is less conclusive for CVC structures.

A potential explanation for the observed discrepancy between CVCV(C) and CVC targets is that the analysis of CVC NAP targets might have been underpowered. While the dataset was substantial—nearly 20,000 tokens overall, with almost 10,000 tokens analyzed after excluding dorsal and liquid consonants—the instances displaying various agreement types in child forms were scarce. This rarity could partly stem from the structural differences in the targets: in C_1_VC_2_ configurations, the second consonant (C_2_), being syllable-final, is more susceptible to deletion than in C_1_VC_2_ V(C) configurations. This likely reduced the probability of observing agreement between C_1_ and C_2_. For CVC targets, the mean rates of agreement were 0.0031 for place-and-manner and 0.0014 for place-only, with an estimated effect size of 0.30. These figures suggest a real difference, although verifying this would require a larger dataset with a higher incidence of relevant examples. However, compared to the means for CVCV(C) targets—0.0299 for place-and-manner and 0.0056 for place-only, with an estimated effect size of 0.42—the magnitude of the observed difference in CVC targets likely represents a smaller effect.

If segment repetition is a response to pressure against remembering and accessing different consonants in a word, there may be memory-based reasons why it is invoked more frequently between C_1_ and C_2_ in C_1_VC_2_ V(C) targets, where both consonants are in syllable-onset positions, than in C_1_VC_2_ targets, where one consonant forms a syllable coda. Memory studies in adults have demonstrated that serial recall improves when repeated elements are positioned similarly within groups. For instance, recall accuracy increases when participants memorize strings of six elements with repetitions located identically within tripartite units (e.g., “12R| 34R,’ where ‘R’ denotes the repeated element, and ‘|’ indicates a pause) (Henson, 1998). In the case of phonological strings, this repetition facilitation should be stronger for onsets than codas in English and other languages with similar syllable structures as, in those languages, syllable-initial sounds are given more weight than other sounds in lexical processing by infants (Jusczyk et al., 1999), toddlers (Swingley et al., 1999), and adults (Creel et al., 2006). Thus, the facilitation of repetition in CVCV(C) target forms over CVC forms might partly be attributed to these positional effects within syllabic structures and their corresponding impact on memory processing.

Age was another potential determinant influencing the prevalence of place-and-manner agreement or segment repetition. Notably, the disparity between place-and-manner and place-only agreement was more pronounced in the 25-word point cross-linguistic dataset, which mostly included word productions from children aged 1;4–1;6, compared to the broader English longitudinal dataset spanning ages from 1;0 to 3;0. Statistical analyses of the longitudinal data revealed a narrowing gap between these types of agreement from the age group 1;0–1;5 to 2;0–2;5. Younger children, particularly those younger than 1.5 years, likely experience greater cognitive challenges in memorizing and retrieving word sounds from their developing lexicon. This increased difficulty may prompt more frequent use of segment repetition as a strategy. Such a pattern aligns with findings in older children aged 4–9 years and adults, who also exhibit significant segment repetition in word recall tasks (Aitchison & Chiat, 1981; Basnak & Ota, 2024; Ota et al., 2021). In short laboratory experiments, participants are similarly engaged in forming early lexical representations, akin to the processes observed in young children during initial vocabulary acquisition. Consequently, if this interpretation of age-related effects holds true, it implies that memory constraints exert a stronger influence on word production up to approximately 1.5 years, significantly shaping the characteristics of reported CCH in this early period. The influence of these effects is likely to diminish as children grow older.

### 4.2 Implications for alternative accounts of CCH

To what extent can these findings be explained by previously proposed accounts of CCH? Descriptively, these patterns can easily be accommodated within a grammar-based model that uses rules or constraints to map the target form with the child form. Specifically, a rule or constraint that enforces total assimilation between syllable onsets could capture this tendency, supplementing the existing rules/constraints proposed for place-only assimilation. This new rule or constraint might also be applicable in explaining related phonological phenomena, such as the “identity effect” discussed earlier. However, explanatory mechanisms for place-only assimilation remain ad hoc solutions to a specific developmental phenomenon with no apparent import for mature phonological systems.

An articulation-based model, such as the A-map (McAllister Byun et al., 2016), offers a potential explanation for these findings by suggesting that children find it easier to articulate pairs of consonants that match in both place and manner at syllable onsets. This assumption is plausible because the ease of articulation might vary depending on the consonant’s position within larger structural units like the syllable. But the degree to which children modify target words in their production cannot be fully attributed to articulatory factors, as has been argued by Vihman, Ota, Keren-Portnoy, Lou, and Choo (2023a). In that study, children learning Mandarin or Japanese were found to be better at producing different C_1_ and C_2_ in C_1_VC_2_ V(C) words than children learning English, Finnish, or French, even though such “variegated” targets are lower in proportion in Mandarin and Japanese. This suggests that the reason why Mandarin- and Japanese-speaking children are more successful at producing variegated words is not because they have more articulatory experience in producing sequences with different consonants. Rather, according to Vihman et al. (2023a), it is due to the smaller syllable inventories of Mandarin and Japanese, which allow children learning these languages to more easily remember recurrent phonological units that can be combined to produce words, including those that contain different consonants. Of course, retention and representation of such phonological units may be aided by vocal practice (Keren-Portnoy et al., 2010), and articulatory experience can also be involved in phonological encoding. But cross-linguistic differences in the production of variegated words suggest that articulatory difficulty alone is unlikely to be the driver for children’s modification of target words containing different consonants.

The “one word, one point-of-articulation” stage, as proposed by Levelt (1994) and Fikkert and Levelt (2008), shares similarities with an account focused on constraints in encoding and retrieval in the sense that it also appeals to representational limits in young children. The model notably posits that early CCH manifests as complete place harmony among consonants and vowels. It predicts, for instance, that coronal consonants should co-occur with front (coronal) vowels, labial consonants with rounded (labial) vowels, and dorsal consonants with back (dorsal) vowels. Low vowels lack place features and can co-occur with any consonant. An examination of the examples given in Tables 8 and 9 reveals many child forms with place-and-manner or place-only agreement that are consistent with this prediction: /ʃip/ *sheep* ↦ [siʃ], /pʊl/ *pull* ↦ [pub], /mɔɹ/ *more* ↦ [mɔm], /tivi/ *TV* ↦ [titi], /bəlun/ *balloon* ↦ [bubu], /pɛn/ *pen* ↦ [dɛn], /tʌmi/ *tummy* ↦ [tʌni]. While these observations are preliminary and require confirmation through more systematic analysis, they suggest that agreement in child forms aligns with the “one word, one place” hypothesis, involving both consonants and vowels. As an extension of the proposal by Fikkert and Levelt (2008) that young children assign phonological features to whole words, Altvater-Mackensen and Fikkert (2015) tested the prediction that early word productions tend to have consonants sharing manner of articulation and found supporting evidence in 1-year-old Dutch and German children. However, they also noted that this effect was independent from place harmony, which suggests that they are not a reflection of complete feature agreement between consonants. Further research is necessary to differentiate the interpretation that the place and manner agreement observed here is due to repetitions of identical consonants and the possibility that it is an interaction of the independent effects of place harmony and manner harmony, as predicted by Fikkert and Levelt (2008) and Altvater-Mackensen and Fikkert (2015).

### 4.3 Remaining issues and conclusions

Overall, the results of this study are in support of the hypothesis that memory constraints play a critical role in shaping CCH as young children learn and access the sounds comprising words they are learning. This proposed mechanism, which emphasizes the effects of string-internal repetition in learning and recalling phonological forms, corresponds with findings from studies on novel word learning in older children and adults, and it is consistent with phonological theories concerning the distinct behaviors of identical versus place-sharing non-identical consonants. However, to advance our understanding of these memory constraints, several aspects require clarification. The hypothesis currently lacks specific details about the nature of these constraints. Are the challenges predominantly in encoding consonant specifications in the mental lexicon? Or do they involve retrieving this information during speech production, or perhaps both? Analyzing variability in the production of individual lexical items might provide insights, as consistent production of non-target-like forms would indicate encoding issues, whereas variability akin to adults’ speech errors might highlight retrieval difficulties.

Further investigation is needed to determine how lexical encoding or retrieval interfaces with the directionality (i.e., the tendency toward regressive/anticipatory assimilation) and the place asymmetry (i.e., the propensity for targeting coronals) frequently reported for CCH. In addition, the relationship between different types of agreement or assimilation in child forms warrants exploration. It was posited that child language production should gravitate toward place-and-manner agreement or segment repetition because these reduce the phonological load required for encoding. However, it remains to be seen whether a similar argument can be made for place-only agreement versus no agreement, regarding the conservation of memory resources. These gaps point to the need for a more nuanced understanding of the interplay between memory constraints and phonological processes in the development of speech.

Another issue warranting further exploration is the relationship between the underlying causes of CCH and the variation in its occurrence among different individuals. As evident from the data here, CCH is not only infrequent overall but also exhibits noticeable individual variation. In the longitudinal English data, the percentage of CCH for CVCV(C) NAP targets between 1;0 and 1;5 ranged from 0.0% to 25.0%, with an average of 8.4%. This finding aligns with earlier research that challenges the common perception of CCH as a widespread phenomenon; as a matter of fact, it is frequent only among certain individuals (Levelt, 2011; Vihman, 1978). Given the suggestion in the current work that CCH is a response to memory limitations during early language development, such variation might primarily reflect differences in children’s overall capacity in phonological memory, with those having more limited capacity to accurately encode phonological information showing higher rates of CCH. However, there is an alternative account of this variation. Extensive work carried out under the whole-word phonology approach suggests that CCH is only one of many ways in which children systematically cope with word forms that present representational challenges (Vihman, 2019). Under this understanding, individual differences in CCH may depend on how children individually manage systematization of phonological forms in the developing productive lexicon. In this context, some children may prefer other strategies, such as deletion of consonants or full repetition of syllables, over CCH. These considerations also suggest that the nature of memory involved in early word learning might be more appropriately characterized as an embodied property that emerges from the interaction of perceptual and motoric experience during speech processing and production, rather than a cognitively encoded store of perceived phonological information (Vihman, 2022).

Thus far, I have not addressed the topic of vowel harmony, the complement of consonant harmony. Unlike major place consonant harmony, a phenomenon that is apparently missing in adult phonology, vowel harmony is fairly common among (mature) languages. It occurs in languages where vowels separated by consonants often share features such as backness, height, and advanced tongue root. Vowel disharmony, in contrast, is uncommon. Despite this clear typological symmetry, the developmental implications of vowel harmony remain under-investigated. A number of studies have examined whether vowel harmony is easier to learn than vowel disharmony, focusing exclusively on adult learners (Martin & Peperkamp, 2020; Pycha et al., 2003; Skoruppa & Peperkamp, 2011). Evidence also suggests that very young infants are capable of detecting vowel harmony patterns, which aid them in segmenting speech (Mintz et al., 2018). However, to my knowledge, there have not been any systematic reports of “child vowel harmony,” except as part of full reduplication, or the repetition of a whole syllable. The absence of child vowel harmony might serve as further evidence that CCH is not primarily driven by co-articulatory effects. If it were, one would expect a similar, or even larger, effect on non-adjacent vowels as well. Nonetheless, it is premature to make that assertion, as the seeming lack of child vowel harmony could partially result from either the variable nature of children’s vowel production or the lack of reliability of transcription of vowels in early speech. I will address the latter point below.

Like most prior research on CCH, this study is methodologically limited by its dependence on transcribed data. As can be seen in Table 2, not all the analyzed data have undergone reliability checks, and the available reliability information reveals a level of disagreement that is not negligible. Furthermore, transcribed data are subject to listener biases. A significant concern is that transcribers may misinterpret or overlook the non-phonemic variation in children’s speech. Some illustrative examples can be drawn from Smith’s (1973) study, which involved transcriptions of his English-learning son’s word productions (though these are not included in this current study for lack of audio recordings). Notably, Smith frequently employed phonetic symbols outside the phonemic inventory of English, such as the velar fricative in [ɣak] *shack* or those unexpected by English phonotactics, like the velar nasal in [ŋok] *knock*. Other transcribers might have transcribed these sounds using phonetic symbols that are phonemically or contextually permitted in English (e.g., [gak] rather than [ɣak]), potentially skewing the analysis toward findings of segment repetition. Such problems are likely to be more serious for vowels, which are even less discrete than consonants. This issue of transcription subjectivity has also been pointed out in adult speech error research, where important phonetic features of errors are often not perceived by listeners (Pouplier & Goldstein, 2005). To address these limitations and gain a better understanding of CCH, future research should incorporate alternative methodologies such as acoustic analysis or ultrasound imaging (refer to Gormley, 2003, for an example of ultrasound analysis of CCH in a single subject).

In summary, this study explored the hypothesis that CCH is partially driven by the memory demands that young children encounter as they learn and access the component sounds of words. Support for this idea is derived from the observation that when attempting to produce words with consonants differing in place and manner—especially in CVCV(C) configurations—children frequently produce forms with identical consonants rather than those matching only in place. This evidence suggests that a significant portion of CCH may be more accurately described as segment repetition rather than merely place agreement, challenging conventional views and highlighting lexical memory as a crucial factor in this phenomenon. While memory constraints may not account for all aspects of CCH, there are reasons to believe that they play a pivotal role in the phenomenon and also illuminate how it may be related to subsequent word learning and adult phonological systems. Thus, this study contributes to resolving one of the long-standing puzzles in child phonology.

The findings underscore the significant role of phonolexical memory in early word production more generally. It is widely recognized that children develop their production ability by forming an inventory of sound patterns from individual words (Cychosz et al., 2021; Dollaghan et al., 1995; Ota & Green, 2013; Vihman, 2022) and the broad phonological structure of their ambient language (Vihman, Ota, Keren-Portnoy, Choo, & Lou, 2023b; Vihman et al., 2023a). Nevertheless, our grasp of how these phonolexical components are entered into memory and accessed during speech production remains rudimentary, particularly regarding the limitations set by the cognitive capacities of young children. The insights offered by this research can be expected to spur further investigations into these dynamics, thereby enriching our understanding of early word production.
